# HAS 1: A natural product from soil-isolated *Streptomyces* species with potent activity against cutaneous leishmaniasis caused by *Leishmania tropica*

**DOI:** 10.3389/fphar.2022.1023114

**Published:** 2022-10-10

**Authors:** Bassel Awada, Maguy Hamie, Rana El Hajj, Ghada Derbaj, Rania Najm, Perla Makhoul, Dima Hajj Ali, Antoine G. Abou Fayad, Hiba El Hajj

**Affiliations:** ^1^ Department of Experimental Pathology, Immunology and Microbiology, Faculty of Medicine, American University of Beirut, Beirut, Lebanon; ^2^ Center for Drug Discovery, American University of Beirut, Beirut, Lebanon; ^3^ Department of Biological Sciences, Beirut Arab University, Beirut, Lebanon; ^4^ College of Medicine, Mohammed Bin Rashid University of Medicine and Health Sciences, Dubai, United Arab Emirates

**Keywords:** soil extract, streptomyces, Leishmania tropica, cutaneous leishmaniasis, natural products, bio-guided fractionation

## Abstract

Cutaneous Leishmaniasis (CL) is a neglected tropical disease, classified by the World Health Organization (WHO) as one of the most unrestrained diseases. The Syrian war and the significant displacement of refugees aggravated the spread of this ailment into several neighboring countries in the Eastern Mediterranean Region (EMR). In Syria, *Leishmania tropica* is identified as one of the most aggressive and endemic identified species, causing localized or generalized lesions, often chronic or relapsing. Pentavalent antimonial drugs are currently used as first line treatment against CL. Nonetheless, these drugs exhibit several limitations, including the repetitive painful injections, high cost, poor availability, and mainly systemic toxicity. Besides, the emergence of acquired parasitic resistance hinders their potency, stressing the need for new therapies to combat CL. Natural products (NPs) epitomize a valuable source in drug discovery. NPs are secondary metabolites (SMs) produced by plants, sponges, or a wide variety of organisms, including environmental microorganisms. The EMR is characterized by its immense biodiversity, yet it remains a relatively untapped area in drug discovery. NPs of the region were explored over the last 2 decades, but their discoveries lack biogeographical diversity and are limited to the Red Sea. Here, we isolated previously uncultured environmental soil-dwelling *Streptomyces sp.* HAS1, from Hasbaya region in southeast Lebanon. When fermented in one of our production media named INA, HAS1 produced a crude extract with significant potency against a clinical *Leishmania tropica* isolate. Using bio-guided fractionation, the bioactive compound was purified and the structure was elucidated by NMR and LC-HRMS*.* Our findings establish NPs as strong candidates for treating *Leishmania tropica* and further dwells on the importance of these natural sources to combat microbial infections.

## Introduction

Cutaneous Leishmaniasis (CL) is a parasitic disease caused by the protozoan *Leishmania* species. CL is classified by the World Health Organization (WHO) as one of the most significant neglected tropical diseases (Cavalli and Bolognesi, 2009), affecting around 12 to 15 million people worldwide, and 600,000 to one million new cases occurring annually (Reithinger, 2016; Torres-Guerrero et al., 2017). Particularly, the last decade witnessed an alarming increase in CL incidence (Bailey et al., 2017). CL is endemic in various areas including the Americas, the Mediterranean basin, the Middle East and Central Asia. In the Eastern Mediterranean (EMR) region, Syria partakes the highest prevalence of CL (Mcdowell et al., 2011). Moreover, the Syrian crisis led to increased frightening numbers of CL (Al-Salem et al., 2016), across Syria itself, and its neighboring countries following the displacement of refugees who fled the war (Saroufim et al., 2014; Sharara and Kanj, 2014; Hayani et al., 2015; Burza et al., 2018; Rehman et al., 2018; Bizri et al., 2021). Clinically, CL is characterized by localized or generalized lesions, often chronic or relapsing, on exposed parts of the body (Sharma et al., 2005; Steverding, 2017; Burza et al., 2018). The severity of these clinical manifestations is variable and depends on the causative species among other factors (Torres-Guerrero et al., 2017). The most prominent etiologic agents of CL in the Middle East region are *Leishmania tropica* (*L. tropica*) and *Leishmania major* (Roberts, 2005; Masmoudi et al., 2013; Fakhar et al., 2016), with a predominance of *L. tropica* among Syrian refugees (Saroufim et al., 2014; Bizri et al., 2021). Various therapeutic approaches exist for CL, with multiple variables affecting the type of applied treatment (Heras-Mosteiro et al., 2017). In addition to physical approaches, remedies can be administered topically, orally, or systemically (Palumbo, 2009; Eiras et al., 2015; Heras-Mosteiro et al., 2017; Briones Nieva et al., 2021). Pentavalent antimonials are the first line of defense against CL, with an extensive use of Meglumine antimoniate (Glucantime) (Esfandiarpour and Alavi, 2002; Palumbo, 2009; Eiras et al., 2015; Briones Nieva et al., 2021). These drugs can be injected intra-lesionally, intramuscularly, or intravenously (Palumbo, 2009; Eiras et al., 2015; Briones Nieva et al., 2021). Nonetheless, they exhibit numerous adverse effects such as their painful repetitive injections and systemic toxicity (Torres-Guerrero et al., 2017; Garza-Tovar et al., 2020). Moreover, several reports documented the emergence of resistant parasites to these therapies (Kazemi-Rad et al., 2013a; Kazemi-Rad et al., 2013b; Mohebali et al., 2019; Ozbilgin et al., 2020). Among the emerging treatments of CL, the potency of an immunomodulatory drug, Imiquimod (Arevalo et al., 2001; Firooz et al., 2006; Arevalo et al., 2007; Miranda-Verastegui et al., 2009) and its analog 1-(3-methoxyphenyl)-Nmethylimidazo [1,2-a]quinoxalin-4-amine (EAPB0503) (El Hajj et al., 2018) was reported. Yet, the use of Imiquimod as first-line therapy and the clinical use of its derivative against CL are still out of reach. Thus, the discovery of new alternative drugs capable of efficiently eliminating CL while inducing minimal side effects is fundamental.

Natural products (NPs), also known as secondary metabolites (SMs), originate from a plethora of organisms including environmental microorganisms and remain an important source in the field of anti-infective drug discovery (Pink et al., 2005; Chin et al., 2006). Indeed, approximately 50% of all antimicrobials discovered over the last 4 decades were NPs, their derivatives, or their mimics (Newman and Cragg, 2020). Among NPs, different examples are reported with anti-leishmanial potency (Gervazoni et al., 2020; Carter et al., 2021). Yet, these NPs were produced from a small pool of bacteria, limiting the opportunity to find novel molecules with specific targets and selective toxicity against *Leishmania* species, particularly *L. tropica* (Zhu et al., 2011; Zhu et al., 2012). Thus, the quest for these molecules against this endemic parasite in the EMR, requires the search for uncultured bacteria. Such microbes can typically be found in untapped geographical locations whose full potential was not yet unraveled (Cordell, 2000; Tulp and Bohlin, 2002; Lefevre et al., 2008). The EMR is a great example of unexploited biodiverse area, where the antimicrobial drug discovery was almost exclusively made at the level of the Red Sea. In this study, we purified NPs synthesized by uncultured environmental bacteria inhabiting the soil of Lebanon, and explored their anti-leishmanial activity against a clinical *L. tropica* isolate from a CL patient.

## Materials and methods

### Soil sample collection and bacterial isolation

Soil samples were collected from Hasbaya (HAS), Koura (KR) and Bchamoun (BCH) specific regions in Lebanon. Each of the isolates was named after the region it originated from and the order of its isolation. For example, HAS1 is the first bacterium isolated from the soil of the Hasbaya region, while BCH 8 and KR 24 correspond to 8th and 24th bacterial isolates from the Bchamoun and Koura regions respectively. Soil samples were collected in a sterile container and transported to the laboratory for further processing. The soil was first dried for 7 days at 37°C. Afterward, 3 g of the dried sample were dissolved in 100 ml of autoclaved distilled water and incubated at 55°C for 30 min. Subsequently, serial dilutions (1/5, 1/10, 1/100, and 1/1,000) of the soil suspension were performed in a final volume of 1 ml. These dilutions, along with the original soil solution, were inoculated on soil agar (30 g dried soil, 18 g bacteriological agar, and 10 g corn starch in 1 L distilled water) or on International *Streptomyces* Project-3 (ISP3) agar (20 g oats, 18 g bacteriological agar, and 2.5 ml ISP3 trace elements in 1 L distilled water, pH = 7.8). The plates were then incubated at 28°C for 14 days. Once colonies started to appear, they were purified *via* continuous subculturing on ISP3 agar. When pure cultures were obtained, bacteria were stored at -80°C in a 50% aqueous glycerol solution.

### Secondary metabolite production and extraction

**FIGURE 1:**
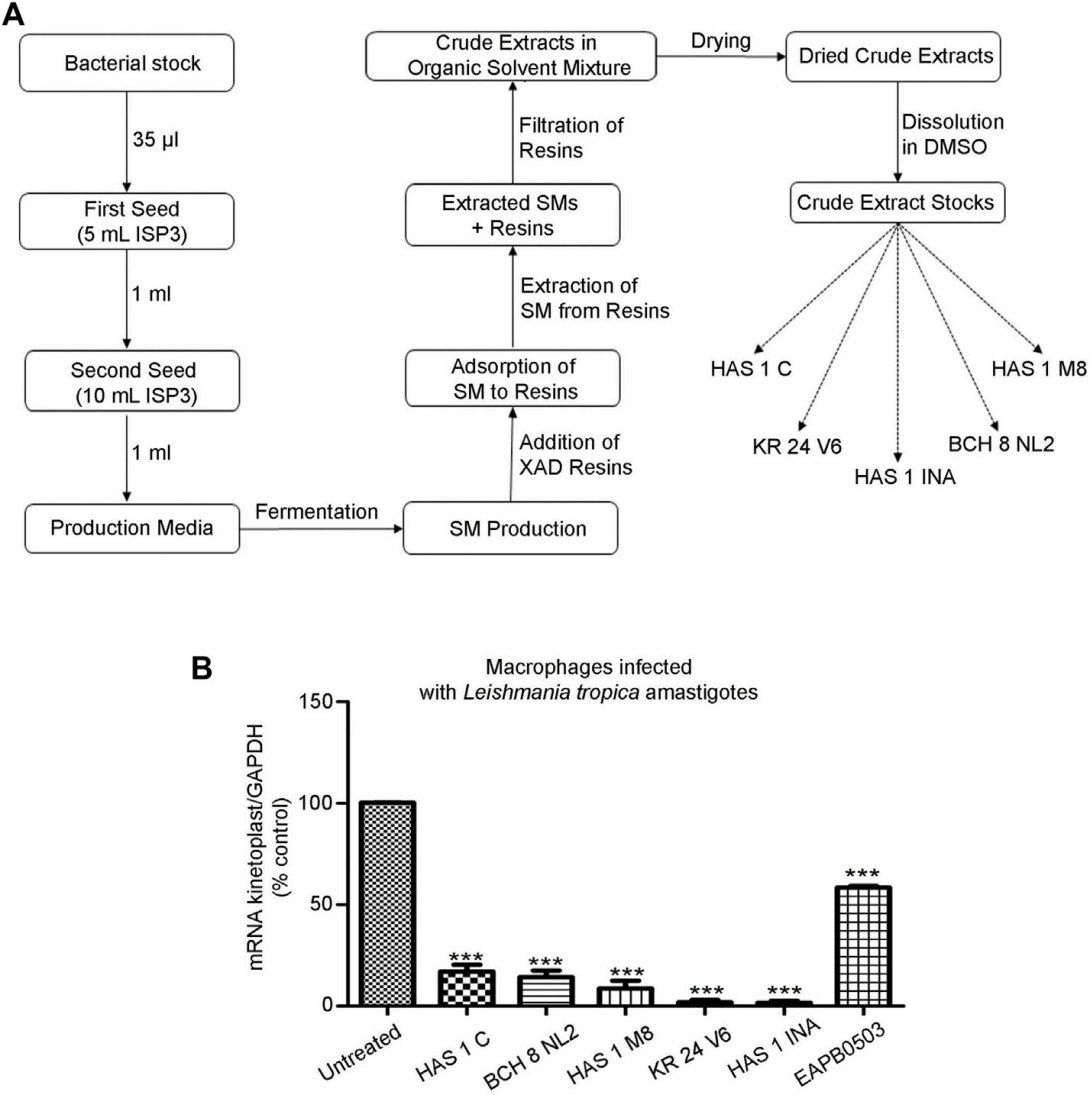
Crude extracts produced by environmental bacteria derived from Lebanese soil inhibit *L. tropica* amastigotes replication. **(A)**. Pipeline of secondary metabolite production by environmental bacteria isolated from soils collected from selected region across Lebanon. Bacteria from Hasbaya (HAS), Koura (KR) and Bchamoun (BCH) were fermented in a collection of 14 different production media. XAD resins were added 14 days post bacterial inoculation to adsorb the SMs, which were then extracted *via* organic solvents. The overall SMs collected from an isolate in a specific medium were named after the isolate followed by the medium name, for example HAS1 INA. **(B)**. Real-time quantitative PCR detection of THP-1 derived macrophages infected with patient-derived *L. tropica* amastigotes treated with 0.01 μg/ml of crude extracts HAS1 C, BCH8 NL2, HAS1 M8, KR24 V6, and HAS1 INA for 24 h. Briefly, THP-1 monocytes were differentiated into macrophages, activated with LPS, and infected with *L. tropica* with a ratio of one parasite/three cells. Treatment with 0.01 μg/ml of the crude extracts or 1 μM of EABP0503 was then performed for 24 h. The results are shown as percentage of untreated infected macrophages. Amastigote transcripts were evaluated by Syber green RT-PCR using kinetoplast specific primers and their percentage of expression was normalized to GAPDH. Results are expressed as percentage of the untreated control (±) SEM and are representative of at least three independent experiments. To validate statistical significance, t-tests were used with *** representing *p*-values < 0.001.

A starter culture, known as the first seed, was initiated by inoculating 35 μl of the bacterial stock in 5 ml of liquid ISP3 (20 g oats and 2.5 ml ISP3 trace elements in 1 L distilled water). This suspension was then incubated at 28°C at 130 rpm for 48 h, before 1 ml was transferred into 10 ml of sterile ISP3 media. This second seed was subjected to identical incubation conditions, after which, 1 ml was used to inoculate 50 ml of each of our 14 different media to induce SM production (Table 1). The cultures were then incubated under the same conditions for 7 days. Then, 1 ml of Amberlite XAD 16N resins was added to each culture and left for 3–4 h to adsorb the SMs. The resin and cell pellets were centrifuged and consequently extracted with methanol. The methanol solution was then defatted with hexane and the layer was evaporated to dryness. The crude was resuspended with ethyl acetate and extracted with 1M HCl. The organic layer was then evaporated to dryness and resuspended in DMSO for further purification by HPLC (Figure 1A). A Phenomenex Luna^®^ 5 μm C18 column 100 Å 250 × 10 mm was used and a gradient from 5 to 95% B in 40 min with (A) H2O+ 0.1% FA and (B) ACN +0.1% FA at a flow rate of 5 ml/min at room temperature. Elution was monitored at 220 and 280 nm. Accordingly, the crude extracts stocks were named HAS1 INA, C and M8 (corresponding to the crude produced by the bacterium HAS1 in medium INA, C and M8 respectively), BCH 8 NL2 (corresponding to the crude produced by the bacterium BCH8 in medium NL2), and KR24 V6 (corresponding to the crude produced by the bacterium KR24 in medium V6).

**TABLE 1:** Composition of the 14 different production media.

Media																					
Components (g/L)		V		Veg		A		B		C		INA	Ra3	GPMY	V6	AF/MS	GYM	M8	NL2	COM
Potato starch														20						
Peptone				5		4							2		5						
Soluble starch		24		20													20	30	
Dextrose		1														20					
Meat extract		3		2		4									5			2		
Yeast extract		5		3		2							4	5	5	2	4	2	2.5	
Malt extract													10	5				10		
Soybean meal				2		2										6				
Glucose												10			20		4	10		25
Triptose		5																		
Maltose						20															
Dextrin						10														
CaCO3				1				0.1		0.1		5				4		3	10	2
Glycerol								20				30	5	20						
*Glycine*								2.5		2.5								4		
Hydrolyzed casein															3				
NaCl								1		1		2			1.5	1				2
KH2PO4								1		1										0.15
FeSO4								0.1		0.1										
MgSO4.7H2O								0.1		0.1										
MgCl2.6H2O													2							
Tween 60										20										
Molasses																			20	
Soy flour																			15	25
Dried beer yeast																				3
Ammonium Sulfate																				2
Soybean Oil																				3
pH		7.2		7		7		7	7		7.3		7.4	7.02	7.05	7.3	7	7	7.8	8.4

### HAS1 isolate characterization

Since extracts of HAS1 isolate exhibited the highest potency against *L. tropica,* HAS1 INA was chosen for further purification. HAS1 was subjected to genomic DNA extraction using the QIAamp DNA Mini Kit (Qiagen). The resulting genomic DNA was used as a template for the preparation of whole-genome sequencing libraries through the Nextera XT library prep kit (Illumina GmbH). The libraries were then sequenced on the Illumina MiniSeq sequencer, 2 × 150 bp. Trimmomatic (Bolger et al., 2014) was used for quality control and trimming of the reads, while assembly was achieved through Unicycler (Wick et al., 2017) on Galaxy. The resulting FASTA file was fed into KmerFinder 3.2 to determine the bacterial species (Larsen et al., 2014). Based on the sequencing results, HAS1 was characterized according to the taxonomic description of the Actinobacteria as follows: first, to visualize the morphology and color of the mycelium, HAS1 was streaked on the ISP3 agar. Second, the isolate was tested for its resistance to sodium chloride (NaCl) through its culture on agar 5,339 (10 g of casein peptone, 5 g of yeast extract, and 20 g of agar in 1 L of distilled water, pH = 7) containing various percentages of NaCl. Third, the isolate’s pH tolerance was investigated by culturing it on ISP2 agar (10 g of malt extract, 4 g of yeast extract, 4 g of glucose, and 15 g of agar in 1 L of distilled water) with different pH levels. For all three tests, the plates were then incubated at 28°C for 10 days before the observation of the growth which was recorded and documented. The biochemical fingerprints of HAS1 were evaluated using the API 20E kit (BioMérieux), according to the manufacturer’s recommendations.

### High performance liquid chromatography mass spectrometry (HPLC-MS)

Measurements were performed using an AB Sciex X500R QTOF ESI mass spectrometer. LC flow was split to 500 nL/min before entering the ion source. Mass spectra were acquired in centroid mode ranging from 150 to 1,000 m/z, resolution R = 30,000. A Luna Omega C18, 150 × 2.1 mm, 1.6 µm column was used, injection volume = 1 µL. A gradient of A) H2O+ 0.1% FA and B) MeCN +0.1% FA at a flow rate of 0.55 ml/min was used to achieve separation. Gradient conditions start at 5% B, increase to 10% B in 1 min, then to 35% B from minute 1→15, then to 50% B from minute 15→22, and finally to 80% B from minute 22→25. After a 1-min hold at 80% B, the system was re-equilibrated for 5 min with the initial conditions. UV data was acquired using a PDA (wavelength 200–800 nm ± 8 nm), MS detection was performed simultaneously.

### *Leishmania tropica* promastigotes culture

*L. tropica* promastigotes were isolated from biopsies of CL patients as described (El Hajj et al., 2018). Sample collection was approved by the Institutional Review Board of the American University of Beirut (PALK.IK.01) (El Hajj et al., 2018). Parasites were maintained in RPMI-1640 (Lonza) supplemented with 10% Fetal Bovine Serum (FBS), 100IU/ml streptomycin/penicillin (Sigma).

### Macrophage culture, infection, and treatment

Human monocytic THP-1 cells (American Type Culture Collection ATCC TIB-202) were seeded at a density of one million cells/well in six well-plates, and grown in RPMI medium supplemented with 10% Fetal Bovine Serum, 1% penicillin-streptomycin, and 1% glutamine. The differentiation of monocytes into macrophages was performed as described (El Hajj et al., 2018). Briefly, the differentiation was induced using 50 ng/ml of phorbol 12-myristate 13-acetate (PMA, Sigma) overnight. The adherent differentiated macrophages were then activated using 1 μg/ml of lipopolysaccharide (LPS) for 4 h. The activated macrophages were subsequently infected with the patient-derived *L. tropica* promastigotes at the ratio of one parasite/3 macrophages. After a 24 h incubation at 37°C, non-internalized parasites were washed out with phosphate buffer saline (PBS). Subsequently, the media was replenished, and a final concentration of 0.01 μg/ml of the crude extracts or the fractions (HAS1 INA Hexane, HAS1 INA chloroform, HAS1 INA Ethyl Acetate, and HAS1 INA Water) or 1 μg/ml of the purified compounds (HAS1 F1, HAS1 F2, HAS1 F3, and HAS1 F4) were added and incubated for 24 h at 37°C. EAPB0503 was used as positive control (El Hajj et al., 2018). The drug was prepared as a 0.1M stock in DMSO and stored at -80°C. Working solutions of 1 μM were freshly prepared in culture media.

### Anti-amastigote activity

After 24 h of incubation with the respective treatments, infected macrophages with amastigotes were washed with PBS, scraped, and collected in microcentrifuge tubes. Tubes were centrifuged at 1,200 rpm for 5 min at 4°C. The supernatant was discarded. The anti-amastigote activity was assessed by quantitative real time PCR and by Immunofluorescence assay.

### Hematoxylin and eosin (H&E) stain

Activated macrophages seeded onto coverslips in six well-plates were subjected to H&E staining. Following infection with *L. tropica* promastigotes at the ratio of one parasite/3 macrophages, and treatment for 24 h with either HAS1-F1 or HAS1-F2, cells were fixed with methanol for 1 min. The coverslips were then covered in Hematoxylin (Fisher Scientific, Canada) and a counterstaining was performed for 30 s before rinsing for 5 min with water. Subsequently, the coverslips were dipped in an alcoholic eosin Y solution (Leica Microsystems, Canada), and rinsed with deionized water before mounting on slides.

### Anti-promastigote activity

To investigate the anti-promastigote activity of the crude extract HAS1 INA and its bioactive fractions (HAS1-F1 and HAS1-F2), a blind counting assay was performed. First, 100 μL per well of *L. tropica* promastigote culture (10^6^ cells/mL) were added in round-bottom 96-well plates. Then, 100 μL of various dilutions of the treatments were added to each well, to obtain final concentrations between 0.01 and 1 μg/ml for HAS1 INA and between 0.5 and 10 μg/ml for HAS1-F1 and HAS1-F2. The plates were then incubated at 25°C for 24 h, then transferred to 37°C for 20 min to reduce parasite motility. The motile promastigotes were then counted using a hemocytometer.

### Quantitative real-time PCR

Total RNA was extracted using the RNeasy Plus Mini Kit (Qiagen). cDNA synthesis was performed using 1 μg of the RNA of each sample as a template, using the iScript cDNA Synthesis Kit (BIO-RAD). Syber green qRT PCR was then completed using the BIORAD-CFX96 machine as described (El Hajj et al., 2018). Primers for amastigote detection target the minicircle kinetoplast DNA (kDNA) (Table 2). Primers for the housekeeping Glyceraldehyde-3-Phosphate dehydrogenase GAPDH, are also listed in Table 2. The PCR cycles included a denaturation step for 3 min at 95°C, followed by 39 cycles of (denaturation for 15 s at 95°C, annealing for 1 min at 57°C, elongation for 30 s at 72°C), and a final elongation for 5 min at 72°C. Percentage of expression was calculated according to the Livak method (Schmittgen and Livak, 2008).

**TABLE 2:** Sequence of primers used in the q-RT-PCR for detection of kinetoplast and the housekeeping gene GAPDH.

Primer	Sequence
Kinetoplast Forward Primer	5′- CCT ATT TTA CAC CAA CCC CCA gT-3′
Kinetoplast Reverse Primer	5′- ggg TAg ggg CgT TCT gCg AAA-3′
GAPDH Forward Primer	5′- CAT ggC CTT CCg TgT TCC TA-3′
GAPDH Reverse Primer	5′- CCT gCT TCA CCA CCT TCT TgA T-3′

### Immunofluorescence microscopy

P6 well plates were seeded with activated macrophages infected with *L. tropica* (1 parasite per three cells) for 24 h and treated with HAS1 F1 or HAS1 F2 for 24 h. After 24 h, coverslips were fixed in 4% paraformaldehyde for 20 min. Permeabilization was performed in Triton (0.2%) for 10 min. Following one PBS wash, a blocking step for 30 min with PBS-10% FBS was performed. A conjugated FITC anti-Leishmania Major Surface Protease (Gp63) monoclonal antibody (CEDARLANE CLP005F) was used at the dilution of 1:1000. Staining of nuclei was performed using 1 μg/ml of Hoechst 33,342 in trihydrochloride trihydrate solution (Invitrogen, H33342) for 5 min. Finally, coverslips were mounted onto slides using a Prolong Anti-fade kit (Invitrogen, P36930). Images were acquired by the Zeiss Axio microscope (Zeiss, Germany) and analyzed using ZEN software.

### Cytotoxicity on PBMCs

To assess the cytotoxicity of HAS1 INA, Has1-F1, and HAS1-F2, trypan blue exclusion assay was performed on human Peripheral Blood Mononuclear Cells (PBMCs). PBMCs were isolated using a Ficoll gradient from left-over peripheral blood specimens collected from the American University of Beirut Medical Center. PBMCs were seeded in 96-cell plates at the density of 200,000 cells/mL in RPMI media supplemented with 20% FBS and 1% penicillin-streptomycin. The cells were then incubated with different concentrations of HAS-F1 or HAS1-F2 (1, 5, or 10 μg/ml) or with 0.01 μg/ml of HAS1 INA for 24 h, and the number of living cells was counted using a hemocytometer.

### Statistical analysis

Graphs were plotted and analyzed using GraphPad Prism. The statistical significance was validated for each treatment using an unpaired *t*-test with the non-treated control. *p*-values < 0.05 were considered as significant. *, **, *** were used for *p*-value < 0.05, <0.01 and <0.001 respectively.

## Results

### Crude extracts from soil isolated bacteria of different Lebanese areas exhibit a potency against a clinical *Leishmania tropica* isolate

The EMR is an unexploited biodiverse area when pertaining to antimicrobial drug discovery. We first collected soil samples from specific regions in Lebanon: Hasbaya (HAS), Koura (KR), and Bchamoun (BCH). The isolation of crude extracts from different soils is described in Figure 1A. The production of secondary metabolites does not only depend on the growth of bacteria, but also on the conditions imposed by the medium and the surroundings. For instance, the carbon source plays an important role for the precursors and energy required for the synthesis of biomass building blocks and secondary metabolites production (Torres-Bacete et al., 2005). Thus, designing an appropriate production medium is an important step in the production of secondary metabolites. The crude extract of isolates from our collection of environmental bacteria from different soil samples were cultured in 14 different production media (Table 1), and tested for their antileishmanial activity, against a *Leishmania tropica* clinical isolate. The antileishmanial effect was assessed by quantifying the expression of *Leishmania*-kinetoplast reflecting the multiplication of amastigotes within macrophages infected with *L. tropica* (El Hajj et al., 2018). Among all tested crude extracts in different production media, five extracts from three soil sources and different production media were able to significantly reduce the kinetoplast transcript levels at a concentration as low as 0.01 μg/ml (Figure 1B), and exhibited a significantly higher potency than EAPB0503, used as positive control (El Hajj et al., 2018) (Figure 1B). While HAS1 C and BCH8 NL2 and HAS1 M8 significantly reduced the multiplication of *L. tropica* amastigotes to less than 20%, KR24 V6 and HAS1 INA almost cleared the parasites (Figure 1B). Notably, HAS1 INA showed the most prominent inhibition of amastigote multiplication with a decline in kinetoplast expression surpassing 98%, when compared to the untreated control (*p*-value < 0.0001) (Figure 1B). Hence the remainder of the present study was based on the bio-guided fractionation of this crude extract to identify novel antileishmanial compounds.

### HAS1 producing strain characterization

**FIGURE 2:**
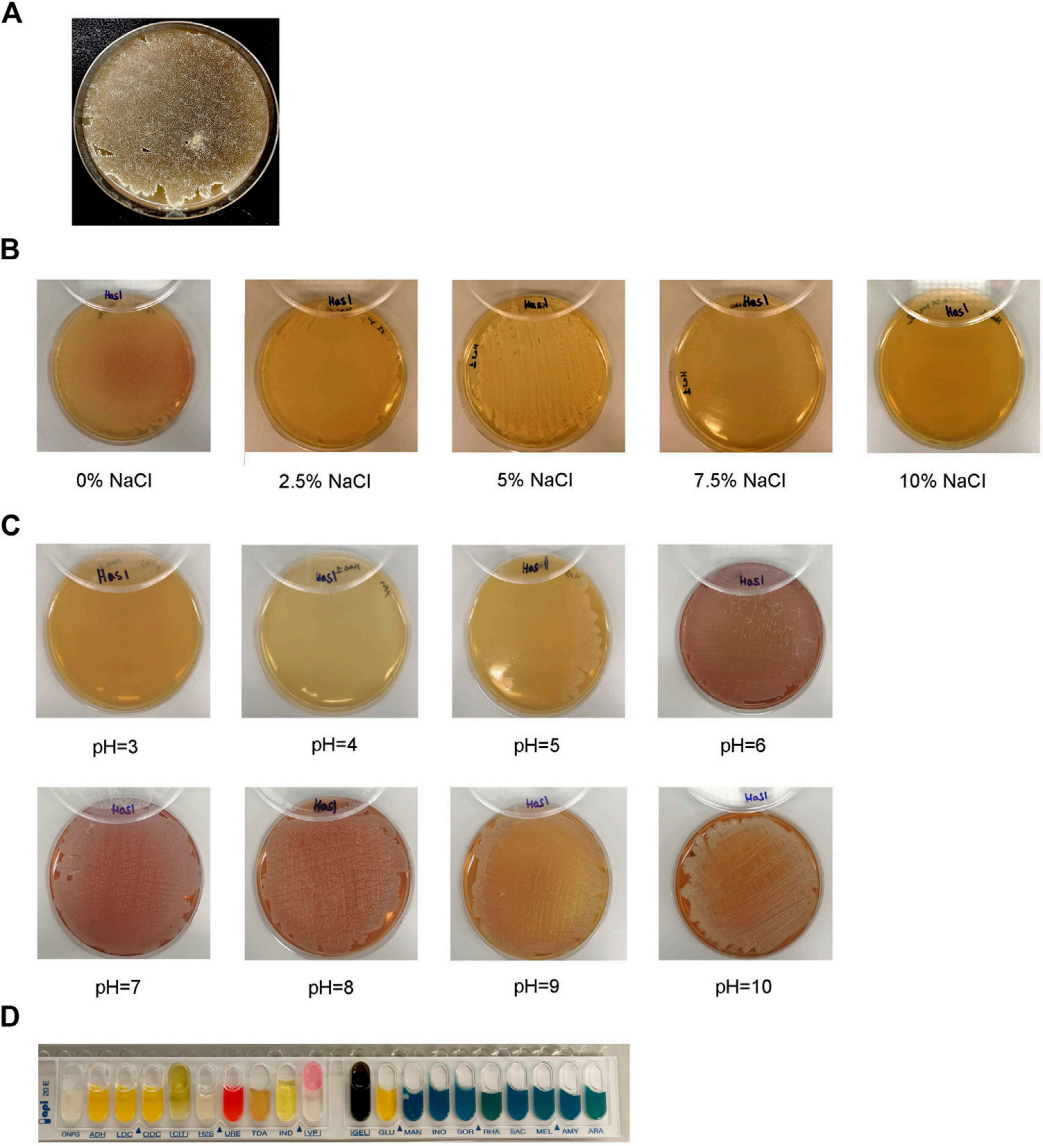
Morphological, physiological, and biochemical characterization of the bacterial isolate HAS1. **(A)**. Growth, shape, color of the mycelium of the isolate HAS1 and spore formation on ISP3 agar 10 days after bacterial inoculation with an incubation at 28°C. **(B)**. Physiological characterization of HAS1 isolate using bacterial growth on the 5,339 agar with different NaCl concentrations (0, 2.5, 5, 7.5 and 10%) after incubation for 10 days at 28°C. **(C)**. Growth of HAS1 on ISP2 agar with different *p*H (3, 4, 5, 6, 7, 8, 9, and 10) after a 10 days at 28°C. **(D)**. Biochemical testing of HAS1 using the API 20E kit.

Using KmerFinder 3.2, HAS1 was most significantly identified as *Streptomyces sp.* CB00271 (q-value = 67,548.74; *p*-value = 1.0e-26). Thus, HAS1 was characterized by standard tests for *Streptomyces*. On ISP3 agar (Figure 2A), HAS1 presented a substrate mycelium with a sand yellow color (RAL 1002) and an aerial mycelium with an oyster white color (RAL 1013). This bacterium formed spores as well. HAS1 was also able to grow normally on the agar 5,339 when the NaCl content was lower or equal to 5%. Whereas minimal growth was observed on the same agar when it contained 7.5% NaCl and no growth was observed at a NaCl percentage of 10 (Figure 2B).

On ISP2 agar, HAS1 showed no growth at *p*H three or 4, while it grew on the same type of agar when the *p*H was superior or equal to 5 (Figure 2C). Finally, using the API 20E kit, HAS1 was able to ferment glucose (GLU-positive) and produce acetoin (VP-positive), and proved to possess both the urease (URE-positive) and gelatinase (GEL-positive) enzymes (Figure 2D).

### HAS1-F1 and HAS1-F2 fractions of HAS1 INA are the bioactive compounds exhibiting the antileishmanial activity against *L. tropica* isolate

**FIGURE 3:**
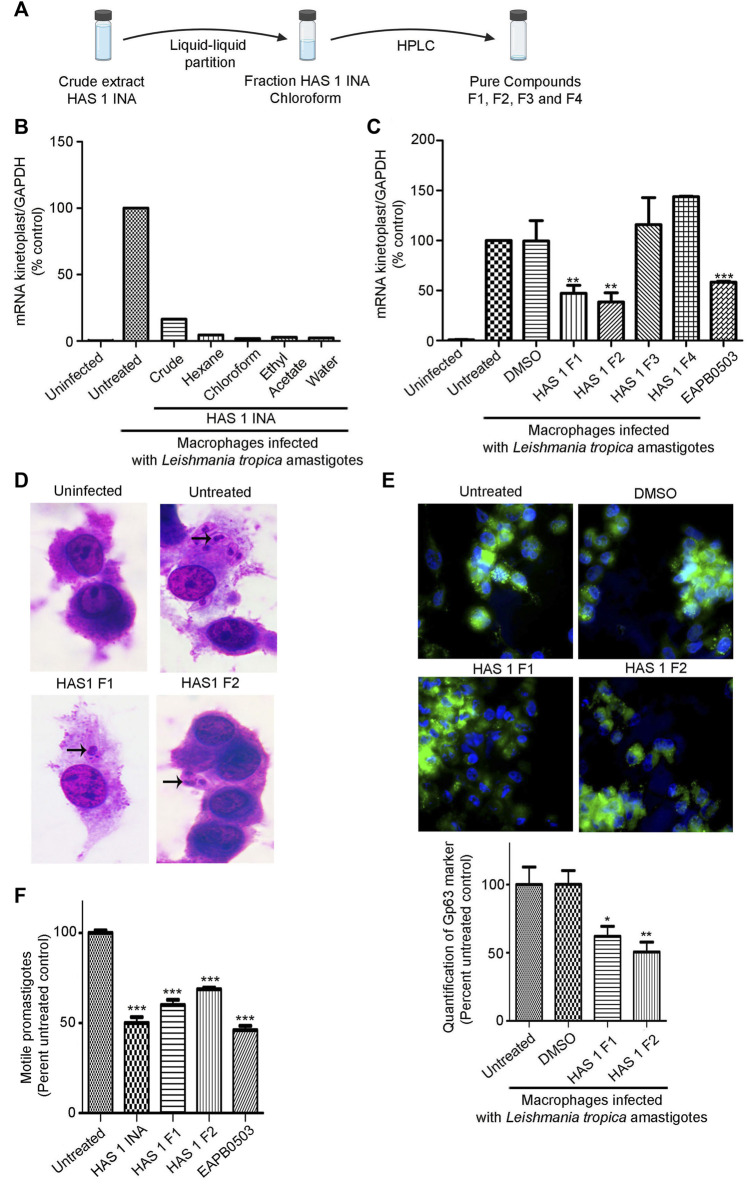
Bio-guided fractionation of the antileishmanial compounds of HAS1 INA. **(A)**. An upscaled fermentation of the HAS1 INA crude was first subjected to liquid-liquid partition generating four fractions: HAS1 INA Hexane, HAS1 INA chloroform, HAS1 INA Ethyl Acetate, and HAS1 INA Water. HAS1 INA chloroform was further fractionated into four pure compounds using HPLC: HAS1-F1, HAS1-F2, HAS-F3, and HAS1-F4. **(B,C).** Real-time quantitative PCR detection of THP-1 derived macrophages infected with patient-derived *L. tropica* amastigotes treated with 0.01 μg/ml of HAS1 INA fractions and the crude extract (B) or with 1 μg/ml of the pure compounds for 24 h or with 1 μM of EABP0503 for 24 h (C). Amastigote transcripts were evaluated by Syber green RT-PCR using kinetoplast specific primers and their percentage of expression was normalized to GAPDH. Results are expressed as percentage of the untreated control (±) SEM and are representative of at least three independent experiments (C) and one representative experiment for (B). To validate statistical significance, t-tests were used with ** representing *p*-values < 0.01, and *** representing *p*-values < 0.001. **(D)**. H&E staining of THP-1 derived macrophages infected with patient-derived *L. tropica* amastigotes treated with 1 μg/ml of HAS1-F1 or HAS1-F2 for 24 h. **(E)**. Immunofluorescence microscopy of THP-1 derived macrophages infected with patient-derived *L. tropica* amastigotes treated with 1 μg/ml of HAS1-F1 or HAS-F2. The Gp63 surface parasite was stained with an anti-Gp63 antibody (green), and nuclei were stained with Hoechst 33,342 (blue). The graph shows the quantification of GP63 as averages from 50 different cells which were then expressed as percentage of the untreated control (±) SEM. To validate statistical significance, t-tests were used with * representing *p*-values < 0.05, and ** representing *p*-values < 0.01. **(F)**. Blind count of motile promastigotes of *L. tropica* treated with 0.01 μg/ml of HAS1 INA, 1 μg/ml of HAS1-F1 or HAS1-F2, or 1 μM of EABP0503 for 24 h. Results are expressed as percentage of the untreated control (±) SEM and are representative of at least three independent experiments. To validate statistical significance, t-tests were used with *** representing *p*-values < 0.001.

The prominent activity of HAS1 INA crude extract prompted us to isolate the bioactive compounds from this mixture. An upscaled fermentation was produced and the SMs were first segregated *via* liquid-liquid partition (Figure 3A). Among the four obtained fractions in Hexane, chloroform, Ethyl Acetate and water, the HAS1 chloroform fraction revealed the most prominent decrease in amastigote multiplication (>98%), at a concentration of 0.01 μg/mL as compared to the untreated control (Figure 3B). Therefore, this fraction was considered for further purification. After HPLC, HAS1 INA chloroform generated four distinct fractions named HAS1-F1, HAS1-F2, HAS1-F3, and HAS1-F4 (Figure 3C). Upon testing their biological activity against *L. tropica* amastigotes, all HAS1 fractions did not result in significant anti-leishmanial activity at the concentration of 0.01 μg/ml. Importantly, at the concentration of 1 μg/ml, HAS1-F1 and HAS1-F2 showed a significant decrease in kinetoplast transcript expression to 47% (*p*-value = 0.0170) and 38% (*p*-value = 0.0029) respectively, as compared to the untreated control (Figure 3C). Importantly, the potency of both fractions was higher than that of EAPB0503, used as positive control (El Hajj et al., 2018) (Figure 3C). The infection of macrophages and the effect HAS1-F1 and HAS1-F2 fractions were then verified by H&E stain (Figure 3D), and quantified using the *Leishmania* Glycoprotein Gp63. Indeed, treatment with 1 μg/ml of HAS1-F1 or HAS1-F2 significantly decreased the percentage of amastigotes, as compared to untreated or DMSO treated controls (Figure 3E). In more details, HAS1-F1 lessened the percentage of intracellular parasites to 62% (*p*-value = 0.0122), while HAS1-F2 reduced the percentage of internalized amastigotes to 50% (*p*-value = 0.0012). As for vehicle control, DMSO did not affect the replication of the intramacrophage amastigotes (Figures 3C,E). Importantly, no cytotoxic effect of either fractions on human peripheral blood mononuclear cells was observed, even at concentrations reaching 5 and 10 μg/ml (Supplementary Figure S1).

We then assessed the potency of HAS1 INA and its bioactive fractions on the promastigote stages of the used *L. tropica* isolate. Interestingly, treatment of promastigotes with HAS1 INA, HAS1-F1, or HAS1-F2 decreased the motile promastigotes count (Figure 3F). Indeed, HAS one INA at the concentration of 0.01 μg/ml exhibited the same potency as EAPB0503 (positive control) and decreased the motile promastigote count to around 50% (*p*-value <0.0001). HAS1-F1 or HAS1-F2, used at the concentration of 1 μg/ml, also exhibited a significant effect on *L. tropica* motile promastigote count, but were less potent than the crude extract or the positive control, and led to decrease of 40% (*p*-value <0.0001) and 32% (*p*-value <0.0001) respectively (Figure 3F). Altogether, our results demonstrate the anti-leishmanial activity of HAS1-F1 and HAS1-F2 bioactive fractions on both the promastigote and amastigotes stages of the tested clinical *Leishmania tropica* isolate.

### Structure elucidation of HAS-1 bioactive fraction F1

**FIGURE 4:**
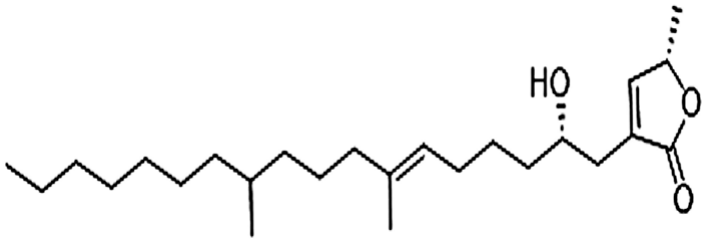
Structure of HAS1-F1.

To elucidate the structure of the two active fractions, HAS1-F1 and HAS1-F2 were run on HPLC-MS (high resolution) in ESI positive mode. HAS1-F1 gave a peak with m/z of 449.3585 for [M + Na] and 427.3779 for [M + H]. The molecular formula generated from these peaks was C_26_H_50_O_4_ with two double bonds. As for HAS1-F2, the peak had an m/z of 393.3443 for [M + H] and a molecular formula of C_25_H_45_O_3_ with four double bonds. While investigating the HPLC-MS of the crude extracts and the various fractions, HAS1-F2 was revealed as a shunt product from biosynthesis, that disappeared overtime when the fermentation was left longer. Hence the possible structure of HAS1-F1 was solely investigated using a Bruker 500 MHz Ascend HD NMR. The elucidation revealed an α-angelica lactone ring and an alcohol on the beta position of the ring (Figure 4). The remaining portion is a fatty acid (Figure 4). This is characteristic to acetogenines that have previously been reported from *Streptomyces sp.* (Taechowisan et al., 2016).

## Discussion

Cutaneous Leishmaniasis remains one of the most important neglected tropical diseases worldwide (Cavalli and Bolognesi, 2009) with an alarming increase in CL incidence reported over the past decade (Bailey et al., 2017). In the EMR, Syria is characterized by the highest prevalence of CL (Mcdowell et al., 2011). The economic deterioration and the war in this country led to a huge displacement of refugees from endemic areas, leading to alarming increases of CL in the neighboring countries including Lebanon (Saroufim et al., 2014; Sharara and Kanj, 2014; Hayani et al., 2015; Al-Salem et al., 2016; Burza et al., 2018; Rehman et al., 2018; Bizri et al., 2021). *L. tropica* is among the most prevalent etiologic agents of CL among Syrian refugees (Saroufim et al., 2014; Bizri et al., 2021).

The treatment of CL still heavily relies on drugs exhibiting a plethora of adverse effects. In that sense, a staple among first-line CL therapies is the pentavalent antimonial Glucantime, which requires excessive repeated parenteral injections inducing localized painful sensation and systemic toxicity (Frezard et al., 2009). Moreover, resistance to Glucantime is emerging, reducing its efficiency in combating CL (Guerin et al., 2002). Second-line drugs against CL such as the antifungal Amphotericin B and the antiprotozoal Pentamidine still pose issues when utilized clinically as they also generate systemic toxicity (Oliveira et al., 2011). Although Miltefosine was recognized as a good candidate for the bedside treatment of CL, evidence from preclinical and clinical studies pointed to the limitations of its use (Monge-Maillo and Lopez-Velez, 2015).

Natural Products were historically, and remain an invaluable source for drug discovery (Newman and Cragg, 2020). This interest in exploiting NPs to create new drug scaffolds has extended to include neglected tropical diseases. Accordingly, leishmaniasis was the target of many studies investigating the inhibitory effects of NPs on its causative species. The majority of NPs with antileishmanial activity originate from plants, namely those which are a part of traditional or folk medicine. Numerous plant-derived crude extracts and fractions revealed their ability to inhibit the replication of *Leishmania sp.* Promastigotes and amastigotes. Using bio-guided fractionation, a variety of structurally diverse antileishmanial pure compounds from NPs were isolated (Rocha et al., 2005; Gervazoni et al., 2020; Carter et al., 2021). In particular, the flavonol Quercetin and the flavonoid Rutin (Mehwish et al., 2019), the sesquiterpene (-)-α-Bisabolol (Corpas-Lopez et al., 2016), Epigallo-catechin 3-O-Gallate (Saduqi et al., 2019) inhibited the proliferation of both promastigotes and amastigotes of *L. tropica*. Furthermore, all these NPs demonstrated lower cytotoxicity levels when compared to the pentavalent antimonial control drug both *in vitro* and *in vivo*. Therefore, NPs offer a vital pool of structurally variable molecules that can compete in both effectiveness and safety with the clinically utilized anti-leishmanials.

The anti-infective efficacy of NPs depended historically on a genus of environmental bacteria known as *Streptomyces* (Antoraz et al., 2015; Quinn et al., 2020). The exploitation of *Streptomyces sp.* in the discovery of novel anti-parasitic agents is typically less common. Nevertheless, these bacteria yielded compounds with important medical efficacy. Ivermectin, a macrocyclic lactone from *Streptomyces avermitilis* is widely used to treat helminth infections (Chabala et al., 1980). The aminoglycoside Paromomycin from *Streptomyces rimosus var. Paromomycinus,* is prescribed for several parasitic infections including leishmaniasis (Wiwanitkit, 2012). We evaluated the anti-leishmanial potential of crude extracts produced by environmental *Streptomyces,* isolated from the understudied niche of the Lebanese soil. The crude extracts produced by *Strepomyces species* from Hasbaya region were the most potent in inhibiting intra-macrophagic *L. tropica* amastigotes *in vitro*. At a concentration as low as 0.01 μg/ml, the crude HAS1 INA caused an almost complete inhibition of the replication of the intracellular amastigotes of an *L. tropia* clinical isolate. This immense activity has not been previously recorded for plants derived extracts tested as antileishmanial compounds against patient-derived internalized *L. tropica* amastigotes. In that sense, the methanolic extract of the leaves of *Myrtus communis* inhibited amastigote replication with an IC_50_ of 40.8 μg/ml (Mahmoudvand et al., 2015), while the alcoholic extract of *Pistacia khinjuk* fruits decreased the growth rate of intramacrophage amastigotes with an IC_50_ of 37.3 μg/ml (Ezatpour et al., 2015).

Following bio-assayed fractionation, two novel compounds HAS1-F1 and HAS1-F2 inhibited the replication of these parasites. Our results follow a recent trend that revolves around screening NPs from *Streptomyces* for activity against *Leishmania* species. Indeed, an extract produced by *Streptomyces sp.* VITBVK2, isolated from a soil sample of a salt pan effectively killed intramacrophage *L. donovani* amastigotes at a dose of 100 μg/ml, and yielded same activity than Miltefosine (Sreedharan and Bhaskara Rao, 2017). Our crude extracts and their bioactive fractions exhibited anti-leishmanial potency 100 to 1,000 folds lower than the extract of VITBVK2. Under the same line, *Streptomyces sp.* isolated from Mediterranean sponges produced the cyclodepsipeptide Valinomycin and the indolocarbazole alkaloid Staurosporine, whose antileishmanial activities were unraveled for the first time. Both compounds inhibited the proliferation of *L. major* promastigotes with IC_50_ values less than 0.11 and 5.3 μM respectively (Pimentel-Elardo et al., 2010). HAS1-F1 and HAS1-F2 inhibited the replication of *L. tropica* amastigotes at doses of 2.3 and 2.5 μM, which are of similar order to that of Staurosporine. Beyond CL, compounds secreted by ants-associated *Streptomyces sp.* inhibited *L. donovani,* the causative agent of another spectrum of leishmaniasis, the fatal visceral leishmaniasis. Indeed, Ortega et al. isolated four known molecules (Mer-A2026B, piericidin-A1, Dinactin, and Nigericin) from three different *Streptomyces sp.* originating from attine ants and reported their antileishmanial activity against *L. donovani* promastigotes (Ortega et al., 2021). Importantly, Dinactin and Nigericin showed superior action than Miltefosine, and were extremely effective in the eradication of the intramacrophage *L. donovani* amastigotes with IC_50_ values of 0.018 and 0.129 μM respectively. Although these compounds are of higher order of activity than HAS1-F1 and HAS1-F2, their cytotoxicity against THP-1 macrophages was extremely elevated (Ortega et al., 2019). The same research group further isolated from the ant-associated *Streptomyces sp.* ISID311, three structurally related macrolides, Cyphomycin, Caniferolide C, and GT-35 which all displayed activity against *L. donovani* promastigotes and intracellular amastigotes (Ortega et al., 2021). Cyphomycin inhibited the replication of intracellular amastigotes with a concentration similar to the HAS1 derived compounds (2.32 μM) while demonstrating low cyctotoxicty. Caniferolide C and GT-35 were more potent (IC_50_ of 0.091 and 0.073 μM respectively) yet showcased intense cytotoxic effects on macrophages.

HAS1-F1 and HAS1-F2 belong to a family of compounds named acetogenins. These polyketide natural products were extensively investigated for their antileishmanial activities. Squamocin and Senegalene were efficient in reducing the proliferation of *L. major* and *L. donovani* promastigotes *in vitro*. The compounds had minimal effective concentrations (MECs), where negative growth was observed, ranging between 25 and 50 μg/ml after 24 h (Sahpaz et al., 1994). Moreover, Squamocin alongside with Rolliniastatine one lysed the promastigotes of several *Leishmania* strains at a dose of 5 μg/ml, similar to the control drug pentamidine (Fevrier et al., 1999). Our results showed around five folds higher efficacy of HAS1-F1 and HAS1-F2, against *L. tropica* amastigotes. Nonetheless, the expression levels of the *Leishmania*-specific kinetoplast markers and its Gp63 decreased significantly upon treatment with either drug as compared to the Untreated infected controls, likely suggestive of an anti-parasitic mode of action. This finding is in line with previously isolated acetogenins such as Rolliniastatin one which limited the replication of both intracellular amastigotes as well as the extracellular promastigotes of *L. donovani* (Raynaud-Le Grandic et al., 2004). Furthermore, four acetogenins isolated from the seeds of *Porcelia macrocarpa* showed anti-leishmanial activity on the amastigotes of *L. infantum*. Among these, the compound (2S,3R,4R)-3-hydroxy-4-methyl-2-(eicos-11′-yn-19′-enyl) butanolide had an IC_50_ of 29.9 μM with no cytotoxic effects on the NCTC cell line (Brito et al., 2022). Although most of the compounds in this family originate from plants, recent evidence shows that *Streptomyces* species can generate acetogenins. In that regard, *Streptomyces sp.* VE2, was able to secrete acetogenins with antibacterial and antioxidant activities (Taechowisan et al., 2016). Therefore, our study is the first to report the isolation of novel acetogenins from a previously uncultured *Streptomyces sp.* HAS1, that were able to inhibit the replication of *L. tropica* amastigotes *in vitro*. Our work marks the first recorded case about establishing a pipeline that screens SMs produced by bacteria against *L. tropica*, the major causative agent of CL in the EMR region, and foreshadows the invaluable opportunities to screen SMs produced by *Streptomyces* for CL treatment.

## Data Availability

The datasets presented in this study can be found in online repositories. The names of the repository/repositories and accession number(s) can be found below: https://www.ncbi.nlm.nih.gov/bioproject/PRJNA873891.

## Supplementary material

Supplementary Figure S1 Cytotoxicity testing of HAS1-F1 and HAS1-F2. Peripheral blood mononuclear cells were incubated with different concentrations of HAS1-F1 or HAS1-F2 (1, 5, or 10 μg/ml) for 24 h. Living cells were counted using the Trypan Blue exclusion dye assay. Results are expressed as percentage of the untreated control (±) SEM are representative of three independent experiments. To validate statistical significance, t-tests were used, and no significance was recorded when comparing any of the treatment groups to the control group.
