# Supplementary figures and images for: HAS 1: A natural product from soil-isolated Streptomyces species with potent activity against cutaneous leishmaniasis caused by Leishmania tropica
Source: Front Pharmacol. 2022 Oct 10;13:1023114. doi: 10.3389/fphar.2022.1023114 (PMC9589300; doi:10.3389/fphar.2022.1023114)

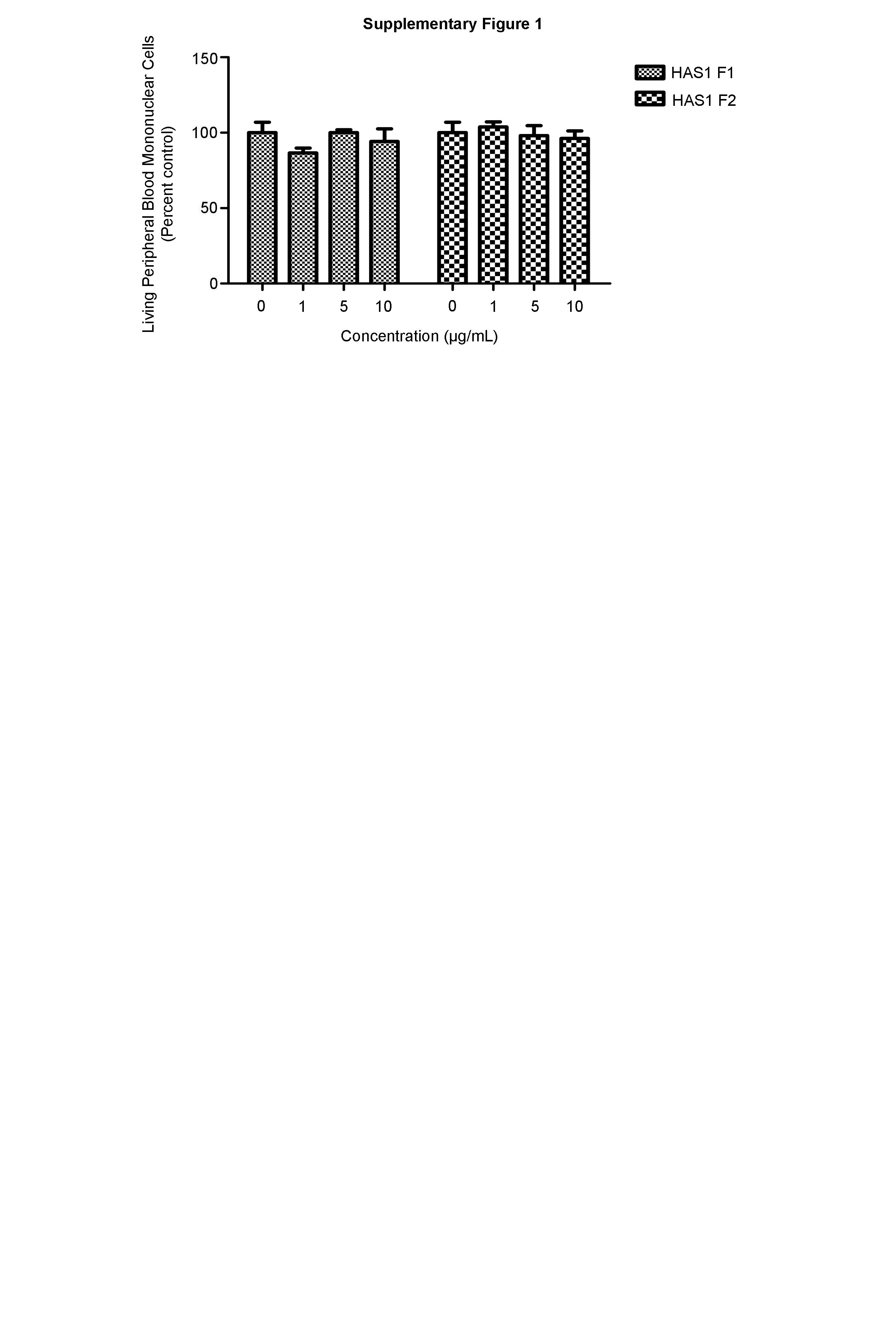

Supplement: Supplementary file 1 [file Image1.JPEG]
